# The Sustainable Development Assessment of Reservoir Resettlement Based on a BP Neural Network

**DOI:** 10.3390/ijerph15010146

**Published:** 2018-01-18

**Authors:** Li Huang, Jian Huang, Wei Wang

**Affiliations:** 1 National Research Center for Resettlement (NRCR), Hohai University, 1 Xikang Road, Nanjing 210098, China; lily8214@hhu.edu.cn (L.H.); 0920805017@hhu.edu.cn (J.H.); 2 School of Public Administration, Hohai University, 1 Xikang Road, Nanjing 210098, China; 3 Business School, Hohai University, 1 Xikang Road, Nanjing 210098, China; 4 College of Harbor, Coastal and Offshore Engineering, Hohai University, 1 Xikang Road, Nanjing 210098, China

**Keywords:** sustainable development assessment, reservoir resettlement, BP neural network

## Abstract

Resettlement affects not only the resettlers’ production activities and life but also, directly or indirectly, the normal operation of power stations, the sustainable development of the resettlers, and regional social stability. Therefore, a scientific evaluation index system for the sustainable development of reservoir resettlement must be established that fits Chinese national conditions and not only promotes reservoir resettlement research but also improves resettlement practice. This essay builds an evaluation index system for resettlers’ sustainable development based on a back-propagation (BP) neural network, which can be adopted in China, taking the resettlement necessitated by step hydropower stations along the Wujiang River cascade as an example. The assessment results show that the resettlement caused by step power stations along the Wujiang River is sustainable, and this evaluation supports the conclusion that national policies and regulations, which are undergoing constant improvement, and resettlement has increasingly improved. The results provide a reference for hydropower reservoir resettlement in developing countries.

## 1. Introduction

As one of the main types of resettlement necessitated by development projects, the resettlement caused by reservoir building is an unavoidable problem in water conservancy and hydropower engineering projects. Changes in reservoir resettlement policies around the world are divided into two phases. Before the 1980s, the general policy was relief compensation. However, after the World Bank’s involuntary resettlement policy (1980) was enacted in the 1980s, the development resettlement policy was proposed, and the long-standing situation in which resettlement relied mainly on governmental assistance gradually changed [1,2]. The first reservoir resettlement to put the policy into practice in China was the Three Gorges Project, which attracted worldwide attention [3]. Later, as China’s hydropower development peaked, particularly through the large-scale hydropower development over the past ten years and the increasing numbers of large- and medium-sized water conservancy projects during the same period, the extensive scope of reservoir resettlement affecting large populations has become noteworthy. Huge differences in the customs and cultures among the communities resettled in different regions forced local governments to actively explore more effective compensation and resettlement measures [4,5]. After years of practice, reservoir resettlement gradually changed from the “blood transfusion” compensation type, which “focuses on relocation while making light of resettlement” and “focuses on submerging compensation while making light of reservoir area development”, to a “hematopoiesis” supporting type, which integrates resettlement into the economic development of the reservoir area and the resettlement area [4,6,7]. With the continuous practice of resettlement development policies, scholars’ focus has also changed gradually from research on compensation standards, resettlement means, relevant policies, and work mechanisms to the evaluation of the resettlement effect, post-placement support and sustainable development [8]. Recently, many scholars have focused on reservoir resettlement evaluation; at the same time, many have discussed the long-term livelihood problems of reservoir resettlement from the perspective of sustainable development [9,10] and, based on the resettlement effect, proposed a series of solutions for the migrants’ livelihood issues [11], such as strengthening their employment skill training, providing technical support for characteristic planting, improving their living and education conditions and providing shop-front spaces.

In synchrony with the practice of reservoir migration, scholars continue to sum up and learn from previous experiences and lessons and to actively explore more reasonable compensation standards and resettlement methods. To gain a more objective and accurate understanding and evaluation of the resettlement effect, many scholars have proposed different resettlement evaluation index systems, but early studies on reservoir resettlement evaluation were based mainly on social evaluation, production and living standards evaluation, economic evaluation, etc. [12,13].

In recent years, as “people-oriented,” “sustainable development,” and other ideas have been proposed, scholars have focused more on long-term livelihoods and sustainable development in reservoir resettlement [14]. Recently, relevant scholars have made achievements in studying long-term livelihoods and sustainable development after the resettlement of reservoir resettlers by tracking investigation [15,16,17], factor analysis [18], and so on. An entirely independent and complete evaluation system has not yet been formed to evaluate the sustainable development of reservoir resettlement. The sustainable development evaluation index system (Sustainability Guidelines and Sustainability Protocol) of the International Hydropower Association (IHA) is used indiscriminately [19]; however, some of its indicators do not match actual aspects of reservoir resettlement, resulting in many difficulties in implementation. Based on the characteristics of different projects, regions and types of resettlement, an evaluation index system for evaluating the sustainable development of reservoir resettlement is constructed in this paper [20,21,22].

In the selection of evaluation methods, the superior advantages of a back-propagation (BP) neural network compared to other evaluation methods are considered [23,24], such as its ability to reduce the influence of subjective factors on the evaluation index and to concurrently handle multiple sets of data and its strong fault tolerance, adaptivity, computer-programmed operation, and high evaluation efficiency. Therefore, to construct an evaluation index system, this paper evaluates the sustainable development of reservoir resettlement based on BP neural network.

The development of the Wujiang River basin in China includes the large-scale construction of many large- and medium-sized hydropower stations, involving hundreds of thousands of migrants and numerous complicated migration problems. At the same time, as Wujiang is the first basin in China in which hydropower station construction has been conducted in a cascade style and through rolling development, in addition to the empirical analysis of the construction of hydropower stations in the Wujiang River basin, this study has important guiding significance and reference value for perfecting the project resettlement theory system and future project resettlement practice. The Wujiangdu, Dongfeng, Hongjiadu, and other hydropower stations are selected as typical cases here, both because we wish to collect comprehensive data and because the construction of these three hydropower stations involved the development of China’s reservoir resettlement policies as they progressed from imperfect to perfect; thus, exploring them has value in several different areas.

## 2. Establishment of an Evaluation Index System for Resettlers’ Sustainable Development

### 2.1. Principles of Index System Building

The first steps in the sustainability assessment of reservoir resettlement involve interviewing sample households of both resettlers and host inhabitants in villages affected by resettlement, collecting statistical data, and conducting a meticulous investigation based on these materials; sustainability is verified by statistics, analysis, and comparison of specific assessment indexes. Therefore, the index system must reflect the levels of production and livelihood and social conditions both before and after resettlement and satisfy the special demands proposed by the project investors and the entities managing the project. The following principles should be observed in selecting indexes.

(1) The System Integrity Principle

This principle refers to the fact that indexes from sample households, villages, counties, and even the entire reservoir and resettlement area form an integral system, and each becomes a factor of the whole system. For example, sample households, villages, counties and the entire reservoir area have grain output indexes; thus, grain output indexes at different levels form a complete system and can be indicated for the whole system.

(2) Data Comparability Principle

This principle refers to the fact that the data obtained in the investigation can satisfy the demands of horizontal and vertical comparisons and show their relation. For example, horizontally speaking, there is compatibility between data for the reservoir resettlers and the affected inhabitants obtained both before and after resettlement; vertically speaking, there is compatibility between data obtained in the reservoir area and in the affected area or in different host sites in the same area.

(3) Feasibility Principle

This principle refers to the fact that all related definitions are clear and accurate, and all data follow the rules of statistics for convenient investigation and analysis. For example, regarding the source of income in households with agriculture as their main income source, in addition to a clear definition of agricultural types, the cultivated area of each crop, per-unit yield, and cost of seed, fertilizer, water, machines, etc., should be accurately and quantitatively defined through a simple and accurate investigation, with statistics determined by monitors and evaluators.

(4) Relativity Principle of Important Indexes

This principle refers to the requirement of a definite relation between the main indexes in the assessment. For example, the Engel coefficient is the ratio of food consumption to all living expenses, and deviation may arise if there is no exact investigation of all foods and living expenses.

(5) Index Effectiveness Principle

This principle refers to the application value of the indexes and their application frequency in monitoring and assessment. It is advisable to choose indexes with a high assessment value and high application frequency as the main indexes and to exclude indexes with lower application frequency, such as the courtyard area and nonproductive slop area of displaced families.

(6) Integrity Principle

This principle refers to the fact that assessment indexes should reflect the main social and economic features at all levels. For example, the per capita net income of the relocated and host populations is an important comprehensive index that can reflect economic and social variations at all levels; therefore, the investigation of this index should be conducted in a meticulous and in-depth manner, with an accuracy level above 90%.

Overall, attention should be paid to all the above-mentioned principles in choosing these indexes; they should reflect all aspects of resettlement and meet the scientific, objective, and just requirements of monitoring.

### 2.2. Index System and Index Interpretation

According to its basic content, the index system of sustainable development assessment for resettlement consists of five aspects: population, economy, resources, environment, and society.

The index of population sustainable development after resettlement consists of the labor force ratio, the aging population rate, and the enrollment rate of school-age children and reflects the population quality and structure. The assessment of resettlement population sustainable development can be conducted in two aspects: population structure and population quality. Population structure is the quantity and proportional relation of all attributive characters of a population, and it usually includes such aspects as age, gender, profession, culture, and urban and rural distribution. Population quality, defining the connotation and extension of a population, includes its ideological, cultural, and physical qualities. Education can be the starting point of population quality assessment, with attention focused on the enrollment of school-age children, the educational background of the labor force and their skill training [25].

The index of economically sustainable development after resettlement consists of nonagricultural employment rate, per capita net income, annual per capita net income growth rate, and food consumption rate, reflecting the resettlers’ employment, income, and consumption. The resettlement economy’s sustainable development may be studied in four aspects: employment, income, consumption, and savings. Means of livelihood refers to people’s way of obtaining food in a different eco-environment and technological level, i.e., the means of livelihood of a certain group. Employment, an important aspect of resettlers’ livelihood, can be counted and assessed by employment time and type, and special attention should be paid to the standby time of the labor force and the nonagricultural employment rate of the agricultural labor force. Related research on income should include the nonagricultural income rate, per capita net income, and the annual per capita net income growth rate. Research on consumption should include the proportion of food consumption in gross expenditure and the sum of a certain type of electrical equipment owned by a certain number of resettlers. Research on savings should begin with per capita savings deposits.

The index of resources’ sustainable development after resettlement consists of savings deposits per capita, per capita food output, paddy rice yield per unit area, and per capita cultivated land and garden plots; it refers to savings, resource input and output, and quantities of resources. Research on resource reserves should focus on the average amount of resources, such as per capita cultivated land, per capita garden plots, per capita staple food output, and staple food yield per unit area. The quality of resources is important; high-quality resources are of great help in enabling people to create value and assets more easily and quickly and in promoting better and faster social and economic sustainable development. The input and output of resources, to a great degree, are affected by resource distribution and utilization. Resource allocation is a specific form of the law of value, and the reasonable allocation and utilization of resources can promote sustainable development of resources.

The index of environmentally sustainable development after resettlement consists of per capita housing space, the steel–concrete structure house rate, and distance to a road, school, and health center, reflecting the situations of housing, infrastructure, and public facilities. The sustainable development of housing after resettlement is embodied mainly in housing conditions, which are studied by the investigation of per capita housing space, house material quality and structure, and housing security, which is examined by investigating the number of dangerous buildings for resettlers in the resettlement area and host households in dangerous locations. Infrastructure is the development foundation of each undertaking of the resettlement economy, including roads, communication, water, gas, and electricity, and social undertakings, such as education, science, health care, and culture.

The index of sustainable development of society after resettlement consists of environmental adaptation, the rate of returning resettlers, communication between relatives, inter-assistance between neighbors and the function of the organization of parties, reflecting psychological adjustment, resettlement integration, and organization health.

The seven indexes of sustainable development of society after resettlement comprehensively consider resettlers’ psychological state, return behavior, harmonious coexistence with the host inhabitants, and the function of host administration organizations after resettlement and provide supporting materials for social security assessment.

The index system of sustainability levels after resettlement is shown in Table 1.

## 3. Assessment Methods for Resettlement Sustainable Development Based on BP Neural Network

Resettlement sustainability assessment is a highly complex process and, at its core, is a judging process. Such early assessment methods as the scoring method, the index system method, the comprehensive index method, the efficiency coefficient method, the multivariate statistics method, fuzzy comprehensive evaluation, gray system evaluation, and the analytic hierarchy process (AHP) were followed by data envelopment analysis (DEA), artificial neural networks (ANNs), etc. [26,27,28]. Recently, assessment methods have become increasingly mathematical, complicated, and multidisciplinary and have gradually become a new kind of borderline science. Assessment of the sustainability state and sustainable development process is a process of building a measurable index system of sustainability; this index helps indicate the sustainability state and whether this mode of development is sustainable. It is difficult to build and assess an index system because of the interaction and cooperation between many social, economic and natural factors, as well as the nonlinearity, the openness, and the dynamics in the sustainable development process. The sustainable development of resettlement is a dynamic process, and its quantitative and qualitative indexes have features and functions of time, space, level, and quantity.

Neural network evaluation, normative and effective, is fit for the analysis of problems with complex information, fuzzy background knowledge, and unclear rules and can reduce artificial uncertainties; the three-layer structure of the BP neural network is usually used to resolve nonlinear problems [29]. Therefore, the three-layer neural network is selected for sustainability assessment in the step hydropower stations along the Wujiang River.

### 3.1. Data Collection and Preprocessing

Resettlement-affected zones of water resources and hydropower projects should be selected with representative times, geographical locations, economic and social environments and normative assessment indexes in the completed resettlement assessment reports of large water projects (such as the Three Gorges, Gezhou Dam, and step hydropower stations along the Wujiang River); the quantized value of their original evaluation results should be taken as the desired output of the neural network, with full consideration of expert opinions and the original assessment.

By dividing the resettlement-affected zone of water resources and hydropower projects into small areas, such as subzones based on different counties, the investigation data are obtained subzone by subzone and used as input values. The assessment of all subzones in the affected area is consistent with the overall evaluations of the original reports, i.e., the same output value; however, the input value of each subzone is different; thus, many samples are formed.

Within such a resettlement-affected zone, the division is conducted according to administration zones, and the lower the level of the administration zone, the better the results will reflect reality and the more samples will be obtained. It is obvious that basic units are overly general if the prefecture-level administration zone is used as the dividing standard, and it is difficult to collect data if the village-level administration zone is taken as the basic unit for analysis. Based on this reasoning, a county-level administration zone is used as a basic analysis zone for data collection, and the step hydropower stations along the Wujiang River are selected as examples.

There is no unified metric in all the indexes in the index system, so it is difficult to make comparisons. In addition, it is necessary to first unify the attributive value of all indexes before conducting a comprehensive assessment, i.e., nondimensionalizing the index attributive value.

### 3.2. The Assessment Model Structure of Resettlers’ Sustainable Development Based on a BP Neural Network

A comprehensive assessment model of the BP neural network with multi-input, multiple hidden units, and a single output unit is built to better use expert assistance and data. In the network, each input node corresponds to a quantitative value for one assessment index, so each output node corresponds to a comprehensive assessment value; the weight and threshold value connecting each neural node are based on experts’ evaluation information, which enables others to learn from the knowledge and experiences of all the experts and to make an objective assessment.

**Figure 1:**
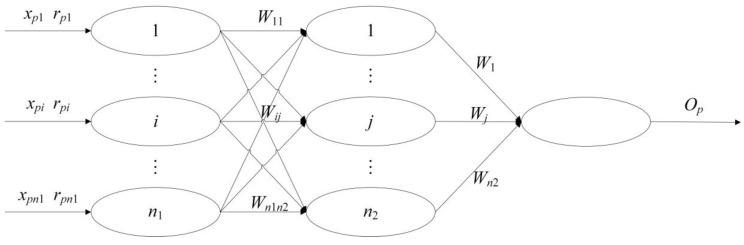
Comprehensive assessment model of the neural network.

The neural network structure [30,31] is shown in Figure 1. In Figure 1, $n_{1}$ and $n_{2}$ are the number of input units and hidden units, respectively, and $x_{p1},\;x_{p2},\cdots ,x_{pn_{1}}$ are the attributive values of sample model p of assessment index $n_{1}$, marked as $x_{p}=\left(x_{p1},\;x_{p2},\cdots ,x_{pn_{1}}\right)$; then, the following attributive value matrix of S sample models is formed (it is presumed that there are S samples).

(1) $$X={\left[x_{1},\;x_{2},\cdots ,x_{s}\right]}^{T}={\left[x_{pi}\right]}_{s\times n_{1}}$$

In Figure 1, $r_{p1},\;r_{p2},\cdots ,r_{pn_{1}}$ are the evaluation vector of $r_{p}$ within the domain of the index set after the relative nondimensionalized function calculation and marked as $r_{p}=\left(r_{p1},\;r_{p2},\cdots ,r_{pn_{1}}\right)$; $W_{ij}$ (i = 1,2,…,$n_{1}$; j = 1,2,…,$n_{2}$) refers to the connecting weight coefficient from unit i at the input layer to unit j at the hidden level; $W_{j}$ (j = 1,2,…,$n_{2}$) refers to the connecting weight coefficient from unit j at the hidden layer to unit i input level; $O_{p}$ is the network output of sample mode p.

With the help of the dimensionless method of index attributive value in resettlers’ sustainable development, the attributive value matrix can be changed to the following assessment matrix: $R={\left[r_{1},\;r_{2},\cdots ,r_{s}\right]}^{T}={\left[r_{pi}\right]}_{s\times n_{1}}$. In the three-layer BP neural network, as shown in Figure 1, the input value should be passed to hidden units; then, the output information from the hidden units is transmitted to output units after function calculation, and the output value is given last. The output value of each unit in the network is calculated according to the following formulas:

(2) $$(1)\;\mathrm{output}\;\mathrm{units}\;O_{pi}=r_{pi}\left(i=1,2,\cdots ,n_{1}\right)$$

(3) $$(2)\;\mathrm{hidden}\;\mathrm{units}\;O_{pj}=f\left(net_{pj}\right)\left(j=1,2,\cdots ,n_{2}\right)$$

(4) $$net_{pj}=\sum_{i=1}^{n_{1}}W_{ij}O_{pi}+\theta_{j}\left(j=1,2,\cdots ,n_{2}\right)$$

(5) $$(3)\;\mathrm{output}\;\mathrm{units}\;O_{p}=f\left(net_{p}\right)$$

(6) $$net_{p}=\sum_{j=1}^{n_{2}}W_{j}O_{pj}+\theta$$

(7) $$f\left(net\right)=\frac{1}{1+\exp\left(-net\right)}$$

where f(net) is the action function of S type, and $\theta_{j}\left(j=1,2,\cdots ,n_{2}\right)$ is the neural threshold value.

## 4. Case Analysis

Guizhou Wujiang Hydropower Development Cooperation Ltd. (Guiyang, China) the first river basin hydropower development cooperation in China, was established in 1992 after approval by the State Council. With the Wujingdu Hydropower Station and the Dongfeng Hydropower Station as its original capital, it started to build and manage seven step hydropower stations, namely, the Hongjiadu Hydropower Station, the Dongfeng Hydropower Station, the Suofengying Hydropower Station, the Wujiangdu Hydropower Station, the Goupitan Hydropower Station, the Silin Hydropower Station, and the Shatuo Hydropower Station, on the mainstream section of the Wujiang River in Guizhou and laid a firm foundation for hydropower development along the river. The construction of these power stations has played an important role in safeguarding regional energy security and promoting the coordinated development of the regional economy, society, and environment.

At present, the construction of these step hydropower stations is almost complete. An assessment and summarization of resettlement caused by these hydropower stations, combined with resettlement practice in the Wujiang River basin, can provide lessons and references for the improvement of resettlement caused by hydropower development projects in other river basins in China, help address the problems that remain during the construction of these hydropower stations, and promote the sustainable development of resettlers and local society.

### 4.1. Sample Selection and Data Collection

Fifty villages were selected as samples, including Linquan, Shiban, Sanhe, and Chabai near the Wujiangdu Power Station, Nayong, Linquan, and Xinren near the Dongfeng Power Station, and Babu, Chadian, and Jinbi near the Hongjiadu Power Station. The following sections describe the main data resources used in this assessment.

(1) Sample Investigation of Displaced Families

The main data resource in the resettlers’ sustainable development assessment is a sample investigation of displaced families. The research group selected 234 displaced families for this investigation and collected 217 valid questionnaires in sample surveys, with extensive coverage in population, resources, production, consumption, and environment.

(2) Data Collected by Resettlement Administration Agencies or Villages

Regarding infrastructure and public facilities, such as hospitals and schools in the assessment, the research group used data collected by local resettlement administration agencies or villages because of their reliability.

(3) Data Released by National Authorities

The research group obtained certain core single indexes and minimum threshold values for the sustainability of core indexes by consulting data released by related national authorities; other data were obtained by calculation. For example, the research group used the rural minimum subsistence level standard released by the National Statistics Bureau of China as the minimum threshold value of sustainability of per capita net income.

In the sustainable development of a typical power station, certain quantitative indexes, such as the annual average income growth rate before and after construction, the annual average consumption growth rate before and after construction, and the employment growth rate before and after construction, are ascertained through quantitative methods, while qualitative indexes are obtained by expert methods.

### 4.2. Ascertaining BP Neural Network Neurons

After the network type of resettlement sustainable development is ascertained in the BP neural network, the number of nodes at each layer should be considered [32].

(1) Ascertaining neuron numbers at the input layer: The output layer is the quantitative data of the assessment index value, and the number of indexes that play a leading role in affecting output value in the assessment is taken as the node number of the input layer; then, the number of the input layer is set at 40 based on the real situation of the hydropower station construction project assessment.
(2) Ascertaining neuron numbers at the hidden layer: The hidden layer number is 25, according to the empirical formula.
(3) Ascertaining neuron numbers at the output layer: There is only one neuron at the output layer because the final result of the resettlement sustainable development assessment is comprehensive.

### 4.3. Neural Network Training and Assessment

**Figure 2:**
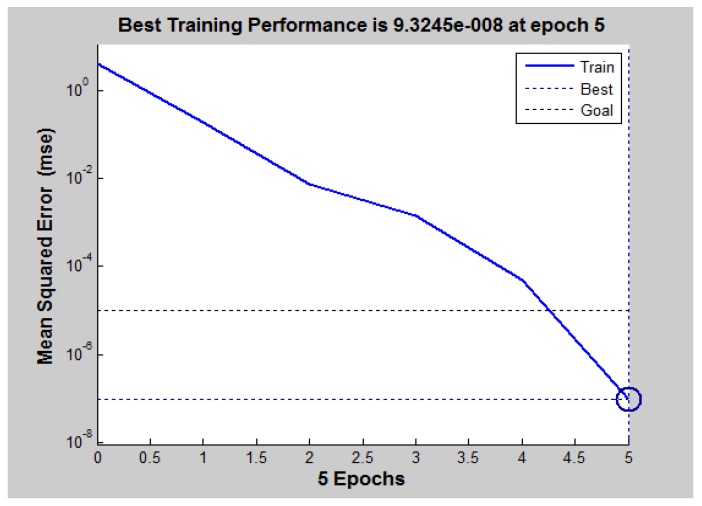
Training curve.

Through the questionnaire survey, the score of comprehensive evaluation of sustainable development of resettlement is determined by the Delphi five-level assignment (1, 2, 3, 4, 5) and then normalized to the value of (0,1), that is, 1, 2, 3, 4, and 5, respectively, corresponding to 0.1, 0.3, 0.5, 0.7, and 0.9. From the 217 × 2 = 434 questionnaires evaluated before and after the relocation, 400 questionnaire data were used in training by the neural network tool of the MATLAB software (MathWorks, Bern, Switzerland). The utility function “train()” was selected for training. Training parameters are established with times set at 1000 and error expectancy value at 0.00001. If the error reached 0.00001 in 1000 times of training, the training is successful; otherwise, it fails, and the training continues until that number is reached. Figure 2 shows the training results. After five times of training, the error expectancy value reached 0.00001, indicating that the training was successful. Then, the remaining 34 questionnaire survey data were used as test samples for testing the trained BP neural network. It was indicated that the neural network is reliable if the error of test result is within 5% [33,34].

In Figure 2, the horizontal ordinates represent training times, and the vertical ordinates represent error expectancy values.

The neural network after training is used in the assessment of three typical power stations: Wujiangdu, Dongfeng, and Hongjiadu. The input data are shown in Table 2, and the comprehensive membership values before and after relocation caused by the Wujiangdu, Dongfeng, and Hongjiadu Power Stations are shown in Table 3.

**Figure 3:**
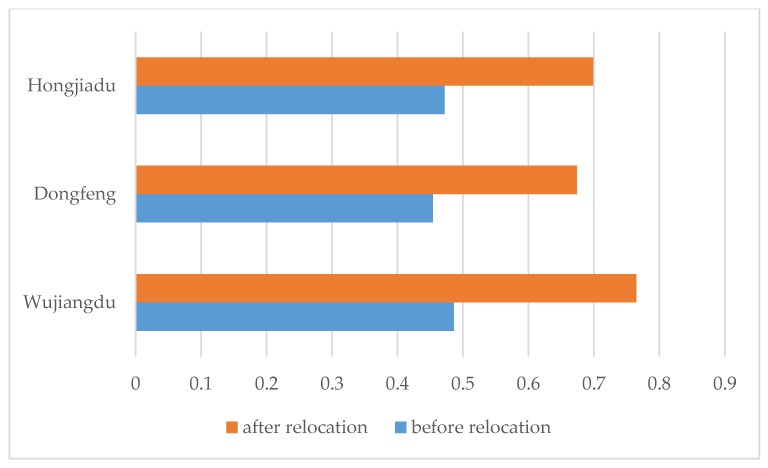
Sketch map of the comprehensive assessment score of the Wujiangdu, Dongfeng, and Hongjiadu Power Stations.

Via the calculation of comprehensive membership and scores in the Wujiang River basin, the membership of each subindex can be calculated individually; finally, the sustainability score of the Wujiangdu, Dongfeng, and Hongjiadu Power Stations is calculated, as shown in Figure 3.

The comprehensive evaluation results show that the overall production and living levels of the Wujiangdu, Dongfeng, and Hongjiadu Power Stations after the relocation were much higher than those before the relocation, and the trend for future production and living standards all move in a better direction. Specifically, the three main indicators that reflect resettlement sustainability—the per capita net income, per capita grain yield, and per capita cultivated land—are all on the rise after the relocation, and each indicator is higher than the minimum value of sustainable development. 

Figure 3 shows that the resettlement sustainability score of the Wujiangdu Power Station is the lowest but with the passing of time, the sustainability of later projects has increased; the reason is that the resettlement caused by different power stations is conducted according to resettlement policies, which are continually being optimized. In view of the long-term development tendency, resettlement caused by step power stations along the Wujiang River is sustainable as a whole. The details are as follows:

(1) Policies and regulations on reservoir resettlement in China are increasingly effective, with improvements based on sound science. The early construction of the Wujiangdu and Dongfeng Power Stations was in the trial stage of China’s reservoir resettlement. Under the historical circumstance, a lack of laws and regulations on reservoir resettlement made much resettlement work lawless. In addition, insufficient attention was given to compensation and resettlement for immigrants when the main concern was construction and economic development. The thought of “heavy engineering, light resettlement” was serious, and the resettlement compensation standards, being strongly administrative, also lacked a scientific basis. With the promulgation and perfection of the State Council Decree No. 74, Decree No. 471 and other policies, the legal and regulatory system taking reservoir resettlement as the main body is becoming more systematic and scientific, providing the legal guarantee for resettlement work.
(2) The number of compensation items for reservoir resettlement is increasing, and the standard is rising; administration by law and people-oriented thinking are organically combined, as are resettlement and new rural construction. Starting from the Hongjiadu Hydropower Station, the compensation for reservoir resettlement began to increase and was gradually refined; the scope of protection was gradually expanded, mainly reflected in the addition of compensation of other attachments added to housing compensation. In contrast, with the deepening of people-oriented thinking, more consideration was given to the future socio-economic development of resettlement areas and the long-term livelihood of immigrants, and more attention was paid to the interests of immigrants when the mode and standards of compensation were determined.
(3) The Wujiang cascade hydropower station has the characteristics of sustainability. In evaluating the sustainability of single power station immigrants and in the comprehensive evaluation of the development of cascade hydropower stations on the Wujiang River, the evaluation results show that the Wujiang cascade hydropower station had the characteristics of sustainability. At the same time, the future development of immigrants showed a growing trend with time. The evaluation results also support the conclusion that, in reservoir resettlement, China’s resettlement policy system is becoming more perfect, the scope of reservoir resettlement compensation more comprehensive, the compensation standard increasingly raised, and the resettlement effect improved.

## 5. Conclusions

This paper contributes to the literature from the following perspectives: (1) This paper develops an evaluation system for resettlers’ sustainable development, including an evaluation index system and an evaluation method system based on a back-propagation (BP) neural network; (2) This paper adopts the evaluation system in China and takes the resettlement necessitated by step hydropower stations along the Wujiang River cascade as an example; (3) The assessment results show that the resettlement caused by step power stations along the Wujiang River is sustainable, and this evaluation supports the conclusion that national policies and regulations, which are undergoing constant improvement, and resettlement are increasingly improving. The results provide a reference for hydropower reservoir resettlement in developing countries.

Generally, policies and regulations for hydropower projects in China are subject to constant improvement. With the initiation and improvement of the Decrees of the State Council of the People’s Republic of China No. 74 and No. 471, the laws regulating hydropower resettlement have gradually become systematic and scientific, providing legal protection for those affected. More offset items are now included in hydropower resettlement, and standards of compensation have increased; thus, resettlement caused by step power stations along the Wujiang River has features of sustainability. Moreover, over time, the sustainability of different power stations has become stronger. This evaluation also supports the conclusion that national policies and regulations are undergoing constant improvement and that resettlement conditions have improved.

To fulfill the goal of building a well-off society in an all-around way in the new era in China, the people’s goals first must be put into practice to resolve problems arising from resettlement, and the following areas should be improved:

(1) Combined resettlement is selected by adjusting measures to local conditions, such as local resources and environment.
(2) Resettlement should be closely combined with national macropolicies, be conducted according to these policies, and keep pace with the times to improve resettlement conditions.
(3) Follow-up support programs should target weak points in a comprehensive assessment for improved effects.

## Figures and Tables

**Table 1:** Index system of sustainability levels after resettlement in the step hydropower stations along the Wujiang River.

Layer of Goals	Layer of Regulations		Layer of Indexes
sustainability levels after hydropower and water resource resettlement	population sustainable development after resettlement	population structure	ratio of labor force in total population
			aging population rate
		population quality	enrollment rate of school-age children
			average years of education of labor force
			annual average time of skill training of labor force
	economy sustainable development after resettlement	employment	annual average standby time of labor force
			nonagricultural employment rate of labor force
		income	per capita net income
			rate of nonagricultural income in total income
			annual income growth rate per capita
			low-income family rate
		consumption	amount of color TVs owned per hundred people
			food spending rate
		savings	per capita savings among resettlers
	resources sustainability after resettlement	efficiency of resources input and output	staple food output per capita
			staple food yield per unit area
			rate of reproduction input in total consumption
		resources quantity	per capita cultivated land and garden plots
			per capita cultivated land (garden plots) with effective irrigation
	environment sustainability after resettlement	housing	per capita living space
			steel-concrete building rate
			rate of households with dangerous buildings among resettlers in resettlement area
			rate of host households in dangerous areas
		infrastructure	rate of rural households with tap water
			rate of rural households with electricity
			shortest distance of residential area to the nearest highway
			rate of residential area with public transportation
		public facilities	average schoolroom area
			distance from residential area to the nearest school
			distance from residential area to the nearest health center
			number of hospital beds per capita within a country
			rate of households with telephone
			shortest distance from residential area to the nearest market
	society sustainability after resettlement	individual psychological adjustment	adjustment to environment in host sites
			return rate among resettlers
		integrity with local society after resettlement	conflict event rate of resettlers in host sites
			communication between relatives
			help from neighbors
		rate of health village organization	village committee organization
			normal function of village committee

**Table 2:** Results of the comprehensive membership calculation of the Wujiangdu, Dongfeng, and Hongjiadu Power Stations.

Subindex	Before or after Relocation	Wujiangdu	Dongfeng	Hongjiadu
labor force rate	before relocation	0.452	0.411	0.338
	after relocation	0.504	0.454	0.366
aging population rate	before relocation	0.05	0.135	0.1
	after relocation	0.055	0.134	0.108
enrollment rate of school-age children	before relocation	0.326	0.241	0.336
	after relocation	0.39	0.31	0.39
average years of education	before relocation	0.266	0.114	0.191
	after relocation	0.306	0.162	0.207
annual average time of skill training	before relocation	0.009	0.014	0.01
	after relocation	0.033	0.027	0.03
annual standby time of labor force	before relocation	0.201	0.117	0.152
	after relocation	0.238	0.257	0.386
nonagricultural employment rate	before relocation	0.131	0.107	0.124
	after relocation	0.364	0.31	0.261
per capita net income	before relocation	0.134	0.154	0.14
	after relocation	0.275	0.35	0.288
nonagricultural income rate	before relocation	0.122	0.038	0.06
	after relocation	0.157	0.11	0.132
growth rate of per capita net income	before relocation	0.114	0.068	0.067
	after relocation	0.142	0.141	0.109
low-income family rate	before relocation	0.034	0.087	0.068
	after relocation	0.054	0.144	0.132
amount of color TVs owned per hundred people	before relocation	0.011	0.008	0.016
	after relocation	0.45	0.448	0.45
food consumption rate	before relocation	0.199	0.279	0.209
	after relocation	0.292	0.193	0.249
per capita savings	before relocation	0.244	0.239	0.098
	after relocation	0.492	0.466	0.15
per capita food output	before relocation	0.111	0.123	0.111
	after relocation	0.273	0.21	0.274
rice yield per unit area	before relocation	0.182	0.105	0.105
	after relocation	0.238	0.285	0.225
reproduction input rate	before relocation	0.111	0.114	0.09
	after relocation	0.05	0.074	0.04
per capita cultivated land and garden plots	before relocation	0.148	0.139	0.379
	after relocation	0.073	0.13	0.309
per capita cultivated land garden plots with effective irrigation	before relocation	0.136	0.145	0.137
	after relocation	0.099	0.104	0.092
per capita living space	before relocation	0.136	0.179	0.151
	after relocation	0.171	0.324	0.348
steel-concrete building rate	before relocation	0.015	0.048	0.021
	after relocation	0.12	0.117	0.12
households living in buildings in dangerous area	before relocation	0.22	0.214	0.216
	after relocation	0.22	0.22	0.22
rate of living in dangerous area	before relocation	0.178	0.174	0.18
	after relocation	0.18	0.18	0.18
households with tap water	before relocation	0	0	0
	after relocation	0.376	0.5	0.357
households with electricity	before relocation	0.021	0.019	0.03
	after relocation	0.17	0.2	0.2
distance of residential area to the nearest highway	before relocation	0.019	0.028	0.08
	after relocation	0.073	0.148	0.148
rate of residential area with public transportation	before relocation	0	0	0
	after relocation	0.071	0.15	0.108
average schoolroom area for each student	before relocation	0.06	0.072	0.049
	after relocation	0.084	0.078	0.087
distance from residential area to the nearest school	before relocation	0.051	0.083	0.078
	after relocation	0.109	0.114	0.103
distance from residential area to the nearest health center	before relocation	0.053	0.048	0.045
	after relocation	0.163	0.176	0.163
per capita number of hospital bed	before relocation	0.09	0.076	0.062
	after relocation	0.181	0.107	0.112
rate of households with telephone	before relocation	0.003	0.004	0
	after relocation	0.049	0.085	0.085
distance to the nearest market	before relocation	0.025	0.026	0.025
	after relocation	0.071	0.067	0.074
adjustment to environment in host sites	before relocation	0.522	0.548	0.556
	after relocation	0.6	0.6	0.6
return rate among resettlers	before relocation	0.4	0.4	0.4
	after relocation	0.4	0.4	0.4
rate of conflict events involving resettlers	before relocation	0.31	0.31	0.31
	after relocation	0.31	0.31	0.31
communication between relatives	before relocation	0.414	0.332	0.28
	after relocation	0.486	0.416	0.336
help from neighbors	before relocation	0.193	0.202	0.194
	after relocation	0.223	0.24	0.232
village committee organization	before relocation	0.45	0.45	0.45
	after relocation	0.45	0.45	0.45
normal function of village committee	before relocation	0.522	0.497	0.539
	after relocation	0.55	0.55	0.55

**Table 3:** Comprehensive membership value before and after relocation caused by the Wujiangdu, Dongfeng, and Hongjiadu Power Stations.

Before or after Relocation	Wujiangdu	Dongfeng	Hongjiadu
before relocation	0.486	0.454	0.472
after relocation	0.765	0.674	0.699
